# COVID-19 Vaccination Coverage, and Rates of SARS-CoV-2 Infection and COVID-19–Associated Hospitalization Among Residents in Nursing Homes — National Healthcare Safety Network, United States, October 2023–February 2024

**DOI:** 10.15585/mmwr.mm7315a3

**Published:** 2024-04-18

**Authors:** David Franklin, Kira Barbre, Theresa A. Rowe, Hannah E. Reses, Jason Massey, Lu Meng, Philip Dollard, Heather Dubendris, Molly Stillions, Lindsay Robinson, Jacques W. Clerville, Kara Jacobs Slifka, Andrea Benin, Jeneita M. Bell

**Affiliations:** ^1^Division of Healthcare Quality Promotion, National Center for Emerging and Zoonotic Infectious Diseases, CDC; ^2^CACI International, Inc, Reston, Virginia; ^3^Goldbelt C6, Chesapeake, Virginia; ^4^Chenega Enterprise Systems & Solutions, LLC, Chesapeake, Virginia; ^5^Lantana Consulting Group, East Thetford, Vermont.

**Summary**

**What is already known about this topic?**

Nursing home residents are at increased risk for severe COVID-19.

**What is added by this report?**

Each week during October 16, 2023–February 11, 2024, 14.9%–26.1% of nursing homes reported one or more SARS-CoV-2 infections. Weekly rates of COVID-19–associated hospitalization ranged from 3.8 to 7.1 per 10,000 nursing home residents. By February 11, 2024, only 40.5% of residents had received an updated 2023–2024 COVID-19 vaccine.

**What are the implications for public health practice?**

During the 2023–24 respiratory virus season, nursing home residents continued to have high rates of COVID-19–associated hospitalization, and up-to-date COVID-19 vaccination coverage remained low. Ongoing surveillance for SARS-CoV-2 infections and COVID-19–associated hospitalizations in this population is necessary to develop and evaluate evidence-based interventions for protecting nursing home residents.

## Abstract

Nursing home residents are at increased risk for developing severe COVID-19. Nursing homes report weekly facility-level data on SARS-CoV-2 infections, COVID-19–associated hospitalizations, and COVID-19 vaccination coverage among residents to CDC’s National Healthcare Safety Network. This analysis describes rates of incident SARS-CoV-2 infection, rates of incident COVID-19–associated hospitalization, and COVID-19 vaccination coverage during October 16, 2023–February 11, 2024. Weekly rates of SARS-CoV-2 infection ranged from 61.4 to 133.8 per 10,000 nursing home residents. The weekly percentage of facilities reporting one or more incident SARS-CoV-2 infections ranged from 14.9% to 26.1%. Weekly rates of COVID-19–associated hospitalization ranged from 3.8 to 7.1 per 10,000 residents, and the weekly percentage of facilities reporting one or more COVID-19–associated hospitalizations ranged from 2.6% to 4.7%. By February 11, 2024, 40.5% of nursing home residents had received a dose of the updated 2023–2024 COVID-19 vaccine that was first recommended in September 2023. Although the peak rate of SARS-CoV-2 infection among nursing home residents was lower during the 2023–24 respiratory virus season than during the three previous respiratory virus seasons, nursing home residents continued to be disproportionately affected by SARS-CoV-2 infection and related severe outcomes. Vaccination coverage remains suboptimal in this population. Ongoing surveillance for SARS-CoV-2 infections and COVID-19–associated hospitalizations in this population is necessary to develop and evaluate evidence-based interventions for protecting nursing home residents.

## Introduction

Nursing home residents are at increased risk for contracting SARS-CoV-2 and developing severe disease compared with community-dwelling older adults (*1*). Staying up to date with recommended COVID-19 vaccination protects nursing home residents against SARS-CoV-2 infection and associated severe outcomes (*2*,*3*). The Centers for Medicare & Medicaid Services (CMS) has required nursing homes to report SARS-CoV-2 infections among nursing home residents to CDC’s National Healthcare Safety Network (NHSN) since May 2020* and COVID-19 vaccination coverage among residents to NHSN since May 2021.^†^ In May 2023, in the context of decreased incidence of SARS-CoV-2 infection and severe COVID-19 disease in the U.S. population, the Public Health Emergency for COVID-19 expired.^§^ To better understand the evolving epidemiology of COVID-19 in nursing home residents, in accordance with mandates from CMS,^¶^ nursing homes began reporting COVID-19-associated hospitalizations among residents to NHSN in June 2023.** In September 2023, CDC’s Advisory Committee on Immunization Practices (ACIP) recommended vaccination with an updated 2023–2024 COVID-19 vaccine for all persons aged ≥6 months (*4*). This analysis used NHSN data to describe rates of incident SARS-CoV-2 infection, rates of incident COVID-19–associated hospitalization, and COVID-19 vaccination coverage among nursing home residents during October 16, 2023–February 11, 2024.

## Methods

### Data Collection

CMS-certified nursing homes report weekly, facility-level data on incident resident SARS-CoV-2 infections, incident resident COVID-19–associated hospitalizations, and resident up-to-date COVID-19 vaccination coverage to NHSN. NHSN defined a case of SARS-CoV-2 infection as a newly positive, laboratory-confirmed SARS-CoV-2 viral test result in a nursing home resident, a COVID-19–associated hospitalization as a hospital admission within 10 days after a laboratory-confirmed SARS-CoV-2 infection,^††^ and up-to-date COVID-19 vaccination as documentation of receipt of an updated 2023–2024 COVID-19 vaccine dose.^§§^

### Data Analysis

Data reported for October 16, 2023–February 11, 2024, were included in the analysis. Facilities missing SARS-CoV-2 infection, COVID-19–associated hospitalization, or vaccination data for a given week were excluded from the analysis for that week. To assess weekly rates of SARS-CoV-2 infection and COVID-19–associated hospitalization, weekly incident counts of SARS-CoV-2 infections and COVID-19–associated hospitalizations and weekly resident counts were used to generate rates of SARS-CoV-2 infection (cases per 10,000 residents) and COVID-19–associated hospitalization (hospitalizations per 10,000 residents), with 95% CIs^¶¶^ for each week. Cumulative weekly SARS-CoV-2 infection and COVID-19–associated hospitalization rates (events per 10,000 residents), overall and stratified by U.S. region,*** were calculated by dividing the cumulative incident SARS-CoV-2 infection and COVID-19–associated hospitalization counts across the study period by the total resident-weeks and multiplying by 10,000. Weekly COVID-19 vaccination coverage estimates (percentage of residents up to date with COVID-19 vaccination) and 95% CIs^†††^ were also calculated. Residents reported to have a medical contraindication to COVID-19 vaccination were subtracted from the denominator for vaccination coverage calculations. Analyses were performed using SAS software (version 9.4; SAS Institute). This activity was reviewed by CDC, deemed not research, and conducted consistent with applicable federal law and CDC policy.^§§§^

## Results

### SARS-CoV-2 Infection

Weekly rates of incident SARS-CoV-2 infection ranged from 61.4 per 10,000 nursing home residents during the week ending February 11, 2024, to 133.8 during the week ending December 3, 2023 (Figure) (Supplementary Table, https://stacks.cdc.gov/view/cdc/153239). The weekly percentage of facilities reporting one or more cases of SARS-CoV-2 infection ranged from 14.9% (week ending October 22, 2023) to 26.1% (week ending January 7, 2024) (Table 1). The weekly percentage of facilities reporting two or more cases of SARS-CoV-2 infection ranged from 8.6% (week ending February 11, 2024) to 16.6% (week ending January 7, 2024). The cumulative weekly SARS-CoV-2 infection rate was 109.3 per 10,000 residents and was highest in the Midwest region (130.1) and lowest in the South (93.1) (Table 2).

**FIGURE:**
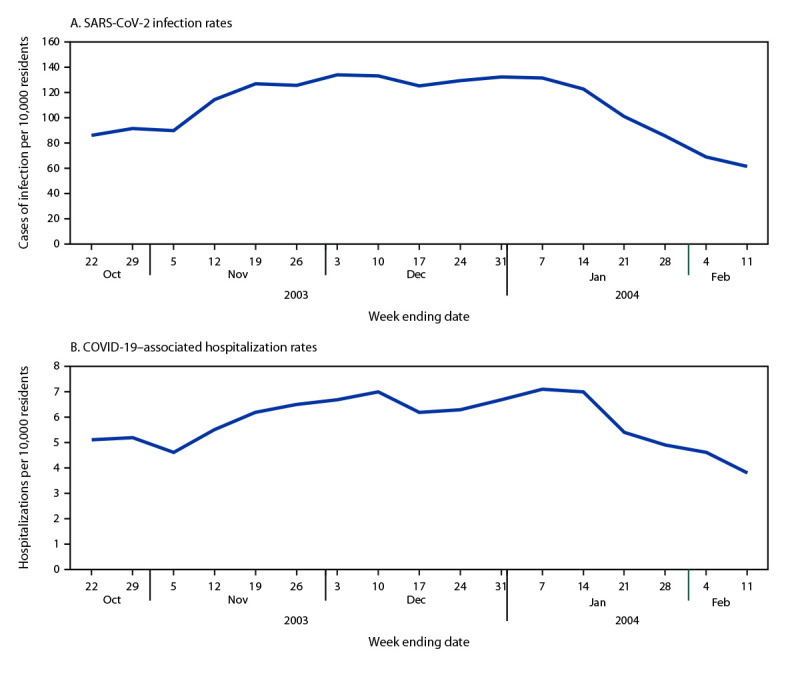
Weekly rates of SARS-CoV-2 infection (A)* and COVID-19–associated hospitalization (B)^†^ among nursing home residents — National Healthcare Safety Network, United States, October 16, 2023–February 11, 2024 * Weekly incident SARS-CoV-2 infections (receipt of a newly positive, laboratory-confirmed SARS-CoV-2 viral test result by a nursing home resident) per 10,000 nursing home residents. ^†^ Weekly COVID-19–associated hospitalizations (a hospital admission within 10 days after a laboratory-confirmed SARS-CoV-2 infection) per 10,000 nursing home residents.

**TABLE 1:** Weekly percentage of nursing homes reporting incident SARS-CoV-2 infections* and COVID-19-associated hospitalizations^†^ and up-to-date COVID-19 vaccination coverage^§^ among nursing home residents — National Healthcare Safety Network, United States, October 16, 2023–February 11, 2024

Year/Week ending	No. of nursing homes^¶^	Total no. of residents	No. of nursing homes with ≥1, ≥2, and ≥5 incident SARS-CoV-2 infections (%)			No. of nursing homes with ≥1 and ≥2 incident COVID-19–associated hospitalizations (%)		% of residents up to date with COVID-19 vaccination (95% CI)**
			≥1 infection	≥2 infections	≥5 infections	≥1 hospitalization	≥2 hospitalizations	
**2023**								
Oct 22	14,637	**1,240,410**	2,188 (14.9)	1,416 (9.7)	745 (5.1)	464 (3.2)	108 (0.7)	16.7 (16.6–16.8)
Oct 29	14,513	**1,232,303**	2,269 (15.6)	1,486 (10.2)	776 (5.3)	485 (3.3)	101 (0.7)	18.5 (18.4–18.6)
Nov 5	14,634	**1,241,181**	2,256 (15.4)	1,506 (10.3)	781 (5.3)	449 (3.1)	96 (0.7)	20.8 (20.7–20.8)
Nov 12	14,619	**1,238,956**	2,561 (17.5)	1,784 (12.2)	987 (6.8)	535 (3.7)	103 (0.7)	23.8 (23.7–23.9)
Nov 19	14,630	**1,240,817**	2,859 (19.5)	1,973 (13.5)	1,075 (7.3)	606 (4.1)	118 (0.8)	26.9 (26.8–27.0)
Nov 26	14,549	**1,226,819**	2,950 (20.3)	2,012 (13.8)	1,065 (7.3)	616 (4.2)	129 (0.9)	28.9 (28.8–29.0)
Dec 3	14,610	**1,233,899**	3,225 (22.1)	2,167 (14.8)	1,097 (7.5)	646 (4.4)	131 (0.9)	31.2 (31.1–31.3)
Dec 10	14,629	**1,240,383**	3,322 (22.7)	2,225 (15.2)	1,174 (8.0)	654 (4.5)	149 (1.0)	33.3 (33.2–33.4)
Dec 17	14,630	**1,242,276**	3,399 (23.2)	2,189 (15.0)	1,078 (7.4)	610 (4.2)	119 (0.8)	35.0 (34.9–35.1)
Dec 24	14,634	**1,241,841**	3,507 (24.0)	2,284 (15.6)	1,119 (7.6)	626 (4.3)	119 (0.8)	36.4 (36.3–36.5)
Dec 31	14,408	**1,211,400**	3,634 (25.2)	2,358 (16.4)	1,080 (7.5)	642 (4.5)	124 (0.9)	37.5 (37.4–37.6)
**2024**								
Jan 7	14,625	**1,234,100**	3,818 (26.1)	2,427 (16.6)	1,076 (7.4)	684 (4.7)	132 (0.9)	37.4 (37.3–37.5)
Jan 14	14,636	**1,244,597**	3,542 (24.2)	2,257 (15.4)	1,017 (6.9)	666 (4.6)	121 (0.8)	37.9 (37.8–38.0)
Jan 21	14,602	**1,247,724**	3,082 (21.1)	1,901 (13.0)	841 (5.8)	555 (3.8)	95 (0.7)	38.5 (38.4–38.6)
Jan 28	14,461	**1,241,081**	2,724 (18.8)	1,604 (11.1)	691 (4.8)	475 (3.3)	89 (0.6)	39.1 (39.0–39.2)
Feb 4	14,529	**1,248,487**	2,432 (16.7)	1,436 (9.9)	539 (3.7)	433 (3.0)	77 (0.5)	40.0 (39.8–40.1)
Feb 11	14,411	**1,240,058**	2,184 (15.2)	1,235 (8.6)	494 (3.4)	380 (2.6)	63 (0.4)	40.5 (40.4–40.6)

* Weekly incident SARS-CoV-2 infections (receipt of a newly positive, laboratory-confirmed SARS-CoV-2 viral test result by a nursing home resident) per 10,000 nursing home residents.

^†^ Weekly COVID-19-associated hospitalizations (a hospital admission within 10 days after a laboratory-confirmed SARS-CoV-2 infection) per 10,000 nursing home residents.

^§^ Up-to-date COVID-19 vaccination was defined as receipt of a 2023–2024 updated COVID-19 vaccine.

^¶^ Nursing homes missing SARS-CoV-2 infection, COVID-19–associated hospitalization, or COVID-19 vaccination data for a given week were excluded from the analysis for that week.

** 95% CIs for vaccination coverage were calculated using Poisson regression models.

**TABLE 2:** Cumulative weekly rates of incident SARS-CoV-2 infection,* COVID-19–associated hospitalization^†^ and percentage up to date with COVID-19 vaccination^§ ^by facility among nursing home residents, by U.S. region^¶^ — National Healthcare Safety Network, United States, October 16, 2023–February 11, 2024

Region	No. of facilities	Resident-weeks	No. of SARS-CoV-2 infections	Cumulative weekly rate of SARS-CoV-2 infection (95% CI)*^,^**	No. of COVID-19–associated hospitalizations	Cumulative weekly COVID-19–associated hospitalization rate^†,^** (95% CI)	% of residents up to date with COVID-19 vaccination (95% CI)^††^
**Overall**	**14,811**	**21,046,590**	**230,105**	**109.3 (108.9–109.8)**	**12,211**	**5.8 (5.7–5.9)**	**40.5 (40.4–40.6)**
Northeast	2,432	4,772,100	54,229	113.6 (112.7–114.6)	2,812	5.9 (5.7–6.1)	47.3 (47.1–47.6)
South	5,508	7,956,877	74,094	93.1 (92.5–93.8)	4,002	5.0 (4.9–5.2)	32.4 (32.2–32.5)
Midwest	4,774	5,619,718	73,134	130.1 (129.2–131.1)	3,782	6.7 (6.5–6.9)	44.7 (44.5–45.0)
Mountain	547	599,880	6,799	113.3 (110.7–116.1)	328	5.5 (4.9–6.1)	41.9 (41.2–42.5)
Pacific	1,550	2,098,015	21,849	104.1 (102.8–105.5)	1,287	6.1 (5.8–6.5)	44.1 (43.7–44.5)

* Weekly incident SARS-CoV-2 infections (receipt of a newly positive, laboratory-confirmed SARS-CoV-2 viral test result by a nursing home resident) per 10,000 nursing home residents.

^†^ Weekly COVID-19–-associated hospitalizations (a hospital admission within 10 days after a laboratory-confirmed SARS-CoV-2 infection) per 10,000 nursing home residents.

^§^ Up-to-date COVID-19 vaccination was defined as receipt of a 2023–2024 updated COVID-19 vaccine.

^¶^ *Northeast*: Connecticut, Maine, Massachusetts, New Hampshire, New Jersey, New York, Pennsylvania, Rhode Island, and Vermont;* South*: Alabama, Arkansas, Delaware, District of Columbia, Florida, Georgia, Kentucky, Louisiana, Maryland, Mississippi, North Carolina, Oklahoma, South Carolina, Tennessee, Texas, Virginia, and West Virginia; *Midwest*: Illinois, Indiana, Iowa, Kansas, Michigan, Minnesota, Missouri, Nebraska, North Dakota, Ohio, South Dakota, and Wisconsin; *Mountain*: Arizona, Colorado, Idaho, Montana, New Mexico, Nevada, Utah, and Wyoming; *Pacific:* Alaska, California, Hawaii, Oregon, and Washington.

** 95% CIs for rates of SARS-CoV-2 infection and COVID–19–associated hospitalization were calculated using mid-p exact tests for incidence density rate.

^††^ 95% CIs for vaccination coverage were calculated using Poisson regression models.

### COVID-19–Associated Hospitalization

Weekly COVID-19-associated hospitalization rates ranged from 3.8 per 10,000 residents (week ending February 11, 2024) to 7.1 (week ending January 7, 2024) (Figure) (Supplementary Table, https://stacks.cdc.gov/view/cdc/153239). The weekly percentage of facilities reporting one or more COVID-19-associated hospitalizations ranged from 2.6% (week ending February 11, 2024) to 4.7% (week ending January 7, 2024) (Table 1). The cumulative weekly COVID-19–associated hospitalization rate was 5.8 per 10,000 residents and was highest in the Midwest (6.7) and lowest in the South (5.0) (Table 2).

### Up-to-Date COVID-19 Vaccination Coverage

Up-to-date COVID-19 vaccination coverage increased from 16.7% to 40.5% over the study period (Table 1). Vaccination coverage as of February 11, 2024, was highest in the Northeast (47.3%) and lowest in the South (32.4%) (Table 2).

## Discussion

During the 2023–24 respiratory virus season, the peak SARS-CoV-2 infection rate (133.8 per 100,000 residents) was lower than the peaks of 306, 435, and 176 during the 2020–21, 2021–22, and 2022–23 respiratory virus seasons, respectively^¶¶¶^; however, SARS-CoV-2 infection continued to cause substantial morbidity among nursing home residents during the 2023–24 respiratory virus season. COVID-19–associated hospitalizations among nursing home residents peaked at 7.1 per 10,000 residents, more than eight times the peak weekly rate of 0.87 per 10,000 among all U.S. adults aged ≥70 years.**** Although data reported to NHSN by nursing homes cannot be directly compared with those submitted by hospitals because of differences in methodology and populations, this stark difference underscores the high risk for COVID-19–associated hospitalization among nursing home residents.

Despite the lower rates of SARS-CoV-2 infection among nursing home residents during 2023–24 compared with previous seasons, during each week of the current study period, 14.9%–26.1% of nursing homes reported one or more incident cases of SARS-CoV-2 infection and 8.6%–16.6% reported two or more incident cases. Although, as of March 2024, CDC no longer recommends that members of the public isolate for 5 days after onset of COVID-19 symptoms,^††††^ this guidance does not apply to residents of long-term care facilities. According to thresholds set by the Council of State and Territorial Epidemiologists and the Council for Outbreak Response: Healthcare Associated Infections and Antimicrobial-Resistant Pathogens, one or more cases of SARS-CoV-2 infection in a nursing home should trigger the facility to conduct additional investigation including, depending on the characteristics of the outbreak and the facility, collecting additional data, conducting additional laboratory testing, implementing infection control practices, and collaborating with relevant public health jurisdictions. A nursing home with two or more cases within 7 days should report the cases to public health, and two or more cases with possible common exposure constitutes an outbreak (*5*). Thus, each week during the 2023–24 respiratory virus season, a proportion of nursing homes underwent case investigations, and some likely experienced SARS-CoV-2 outbreaks.

In September 2023, ACIP recommended the updated 2023–2024 COVID-19 vaccine for persons aged ≥6 months (*4*). During the 2023–24 respiratory virus season, coverage with the updated 2023–2024 COVID-19 vaccine among residents of nursing homes reporting to NHSN reached 41.5% overall and remained <50% in every U.S. region. This finding indicates that an important prevention tool is being underutilized in this population. In February 2024, CDC and ACIP recommended that all adults aged ≥65 years receive 1 additional dose of an updated 2023–2024 COVID-19 vaccine at least 4 months after the previous updated dose; additional doses are also available for persons who are moderately or severely immunocompromised.^§§§§^ Surveillance of COVID-19 vaccination coverage among nursing home residents is critical to supporting tailored outreach activities to increase vaccination coverage.

NHSN is the only national surveillance system continuously monitoring COVID-19 incidence, COVID-19–associated hospitalization, and COVID-19 vaccination coverage among nursing home residents. COVID-19 surveillance data from NHSN is provided to state and local health departments; these data have also been used to support infection prevention and control policy and evaluate vaccine effectiveness (*2*).

### Limitations

The findings in this report are subject to at least four limitations. First, data are reported by nursing homes; therefore, misclassification of SARS-CoV-2 infection, COVID-19–associated hospitalization, and COVID-19 vaccination status of residents is possible. Second, this analysis was conducted using aggregate, facility-level data reported to NHSN; therefore, crude rates included in this analysis could not account for potential person-level confounding factors, including time since vaccination, previous infection, age, or comorbidities. Third, this analysis did not account for regional or facility-level differences in SARS-CoV-2 testing. Finally, COVID-19–associated hospitalization was defined by NHSN as a hospital admission within 10 days after a laboratory-confirmed SARS-CoV-2 infection. Thus, it is possible that some hospitalizations were classified as COVID-19–associated but were the result of other medical conditions. However, NHSN’s method for defining COVID-19–associated hospitalizations is consistent with that of other surveillance systems.^¶¶¶¶^

### Implications for Public Health Practice

COVID-19 continues to cause substantial morbidity among nursing home residents. Nursing homes should continue to implement recommended infection prevention and control practices,***** including encouraging residents, caregivers, and nursing home staff members to remain up to date with all recommended COVID-19 vaccine doses to limit the introduction and spread of SARS-CoV-2 infection within nursing homes (*4*). Ongoing surveillance for SARS-CoV-2 infections and COVID-19–associated hospitalizations among nursing home residents is necessary to develop and evaluate evidence-based interventions for protecting nursing home residents.

## Footnotes

* https://www.cms.gov/files/document/qso-20-29-nh.pdf

†
https://www.cms.gov/files/document/qso-21-19-nh.pdf

§
https://www.hhs.gov/about/news/2023/02/09/fact-sheet-covid-19-public-health-emergency-transition-roadmap.html

¶
https://www.federalregister.gov/documents/2021/11/09/2021-23993/medicare-and-medicaid-programs-cy-2022-home-health-prospective-payment-system-rate-update-home#p-amd-22

**
https://www.cdc.gov/nhsn/pdfs/covid19/archive/ltcf/57.144-form-v14.pdf

††
https://www.cdc.gov/nhsn/pdfs/covid19/ltcf/57.144-toi-508.pdf

§§ NHSN defines up-to-date COVID-19 vaccination for surveillance purposes at the start of each quarter; https://www.cdc.gov/nhsn/pdfs/hps/covidvax/UpToDateGuidance-508.pdf (Accessed April 12, 2024).

¶¶ 95% CIs for rates of SARS-CoV-2 infection and COVID-19-associated hospitalization were calculated using mid-p exact tests for incidence density rate.

*** *Northeast*: Connecticut, Maine, Massachusetts, New Hampshire, New Jersey, New York, Pennsylvania, Rhode Island, and Vermont; *South*: Alabama, Arkansas, Delaware, District of Columbia, Florida, Georgia, Kentucky, Louisiana, Maryland, Mississippi, North Carolina, Oklahoma, South Carolina, Tennessee, Texas, Virginia, and West Virginia; *Midwest*: Illinois, Indiana, Iowa, Kansas, Michigan, Minnesota, Missouri, Nebraska, North Dakota, Ohio, South Dakota, and Wisconsin; *Mountain*: Arizona, Colorado, Idaho, Montana, New Mexico, Nevada, Utah, and Wyoming; *Pacific*: Alaska, California, Hawaii, Oregon, and Washington.

††† 95% CIs for vaccination coverage were calculated using Poisson regression models.

§§§ 45 C.F.R. part 46.102(l)(2), 21 C.F.R. part 56; 42 U.S.C. Sect. 241(d); 5 U.S.C. Sect. 552a; 44 U.S.C. Sect. 3501 et seq.

¶¶¶
https://www.cdc.gov/nhsn/covid19/ltc-report-overview.html#anchor_1594393304 (Accessed April 12, 2024).

****
https://covid.cdc.gov/covid-data-tracker/#new-hospital-admissions (Accessed April 12, 2024).

††††
https://www.cdc.gov/media/releases/2024/p0301-respiratory-virus.html

§§§§
https://www.cdc.gov/vaccines/acip/recommendations.html (Accessed April 12, 2024).

¶¶¶¶
https://www.cdc.gov/coronavirus/2019-ncov/covid-data/covid-net/purpose-methods.html

*****
https://www.cdc.gov/coronavirus/2019-ncov/hcp/infection-control-recommendations.html

## References

[1] Resciniti NV, Fuller M, Sellner J, Lohman MC. COVID-19 incidence and mortality among long-term care facility residents and staff in South Carolina. J Am Med Dir Assoc 2021;22:2026–2031.e1. 10.1016/j.jamda.2021.08.006 34481792 PMC8364806

[2] Wong E, Barbre K, Wiegand RE, Effectiveness of up-to-date COVID-19 vaccination in preventing SARS-CoV-2 infection among nursing home residents—United States, November 20, 2022–January 8, 2023. MMWR Morb Mortal Wkly Rep 2023;72:690–3. 10.15585/mmwr.mm7225a4 37347711 PMC10328477

[3] Dubendris H, Reses HE, Wong E, Laboratory-confirmed COVID-19 case incidence rates among residents in nursing homes by up-to-date vaccination status—United States, October 10, 2022–January 8, 2023. MMWR Morb Mortal Wkly Rep 2023;72:95–9. 10.15585/mmwr.mm7204a3 36701262 PMC9925132

[4] Regan JJ, Moulia DL, Link-Gelles R, Use of updated COVID-19 vaccines 2023–2024 formula for persons aged ≥6 months: recommendations of the Advisory Committee on Immunization Practices—United States, September 2023. MMWR Morb Mortal Wkly Rep 2023;72:1140–6. 10.15585/mmwr.mm7242e1 37856366 PMC10602621

[5] Epson E. Proposed investigation/reporting thresholds and outbreak definitions for COVID-19 in healthcare settings. Atlanta, GA: Council of State and Territorial Epidemiologists; 2023. https://www.corha.org/wp-content/uploads/2024/01/COVID-19-HC-Outbreak-Definition-Guidance_January-2024.pdf

